# Analyzing and mitigating (with LLMs) the security misconfigurations of Helm charts from Artifact Hub

**DOI:** 10.1007/s10664-025-10688-0

**Published:** 2025-07-04

**Authors:** Francesco Minna, Fabio Massacci, Katja Tuma

**Affiliations:** 1 Afdeling Informatica, Vrije Universiteit, Amsterdam, Netherlands; 2 Dipartimento di Ingegneria e Scienza dell’Informazione, Università di Trento, Trento, Italy; 3 Dept. of Mathematics and Computer Science, Eindhoven University of Technology, Amsterdam, Netherlands

**Keywords:** Helm charts, Misconfigurations, Mitigations, LLM, Kubernetes

## Abstract

Helm is a package manager that allows defining, installing, and upgrading applications with Kubernetes (K8s), a popular container orchestration platform. A Helm chart is a collection of files describing all dependencies, resources, and parameters required for deploying an application within a K8s cluster. This study aimed to mine and empirically evaluate the security of Helm charts, comparing the performance of existing tools in terms of misconfigurations reported by policies available by default, and measuring to what extent LLMs could be used for removing misconfigurations. For these reasons, we proposed a pipeline to mine Helm charts from Artifact Hub, a popular centralized repository, and analyze them using state-of-the-art open-source tools like Checkov and KICS. First, the pipeline runs several chart analyzers and identifies the common and unique misconfigurations reported by each tool. Secondly, it uses LLMs to suggest a mitigation for each misconfiguration. Finally, the LLM refactored chart previously generated is analyzed again by the same tools to see whether it satisfies the tool’s policies. We also performed a manual analysis on a subset of charts to evaluate whether there are false positive misconfigurations from the tool’s reporting and in the LLM refactoring. We found that (i) there is a significant difference between LLMs, (ii) providing a snippet of the YAML template as input might be insufficient compared to all resources, and (iii) even though LLMs can generate correct fixes, they may also delete other irrelevant configurations that break the application.

## Introduction

Kubernetes (K8s) has become the de-facto standard tool to orchestrate container-based applications in the cloud, reported to be used by 64% of organizations in production environments from the 2022 Cloud Native Computing Foundation (CNCF) annual survey (Cloud Native Computing Foundation 2022). Helm is a package manager which is used to help deploy applications on K8s clusters. It allows defining applications as charts, a set of folders and files that bundle all resources and dependencies needed by an application, reducing the need to manually write and manage K8s YAML files.

These charts often contain different types of misconfigurations (Johnson 2021; Rahman et al. 2024), and several static analysis tools have been developed to detect them, such as Checkov (PrismaCloud 2024), Datree (Datree 2024), and KICS (Checkmarx 2024), to name a few. Based on rule-based policies, these tools analyze Helm charts and K8s YAML files and highlight security risks, such as missing memory limits or an over-privileged security context for a container. Such tools can detect configuration lines in a YAML file, such as the allowPrivilegeEscalation: true option, that would allow a container to escalate privileges during execution, which is considered a bad practice, according to the K8s CIS Benchmarks^1^. However, even if some of these misconfigurations may seem trivial to detect, they are still very present in the wild; additionally, other options, such as wrong network policies, may not be trivial to detect.

For example, a blog post by Checkov developers describes the number and type of misconfigurations reported by Checkov on several charts (Johnson 2021). For such misconfigurations, there exist several rule-based policies (e.g., “Prevent containers from escalating privileges” — allow Privilege Escalation: false), that check whether a YAML key is present or not, and eventually its value.

Existing tools have different policies and different configurations may satisfy the same policy; however, most rule-based tools do not suggest or implement any mitigations for the corresponding misconfigurations.

Large Language Models (LLM), such as Google Gemini (Google 2024) and GPT (OpenAI 2024), allow, among other things, to write, understand, and refactor source code. Therefore, we are interested in evaluating whether LLMs could be used to refactor a K8s deployment file by implementing new safer configurations. Since LLMs can generate wrong and inconsistent results, for instance, due to flawed training data sources (Huang et al. 2023), we are also interested in measuring their reliability to determine whether they can be used to generate secure cloud configurations. Indeed, a recent study has found that LMM-assisted users produced critical security bugs at a rate no greater than 10% more compared to users coding without LLMs (Sandoval et al. 2024).

This study aimed to mine and empirically evaluate the security of Helm charts, in terms of misconfigurations, compare the performance of existing tools, and measure to what extent LLMs could be used for removing misconfigurations. In doing so, we investigated what is the general status of charts available on Artifact Hub, for example, in terms of the number of misconfigurations reported. We were also interested in what policies are available by default in each tool, and whether there are unique policies or, in other words, if certain misconfigurations can only be detected by one tool. At the same time, we also investigated if and how Large Language Models (LLM) can be used to remove misconfigurations, and whether the implemented mitigations satisfy the tool policies. This study is important and relevant to cloud and LLM practitioners because it sheds light on the current security status of the largest open-source Helm chart repository, it provides a comparison of existing tools to detect cloud misconfigurations, and an extensive evaluation of whether LLMs are effective in sanitizing cloud misconfigurations.

### Implications of Findings

We hereby briefly present the findings of this paper and the corresponding implications for cloud developers and researchers. In terms of misconfigurations (RQ1), we found that the most common are either very environment-dependent (e.g., namespace limitrange and resource quota, and network policies), thus it is difficult to provide a general definition and corresponding fix, or well known challenges, such as defining AppArmor and SecComp profiles, with all and only the needed permissions. This suggests that, even though LLMs can not help in defining lists of permissions (e.g., an AppArmor profiles), because they require running the container or parse the entire application’s source code, they can still provide useful suggestions for environment dependent configurations. In terms of LLM refactorings (RQ2), we found that Claude and GPT provide an answer more often, even though this is not related to the answer’s correctness, and GPT is the LLM satisfying the tools the most. However, GPT is not consistently the best for the most common misconfigurations, thus several models should be queries to get more suggestions; also, more attention should be paid on misconfigurations that require external objects, other than changing few lines in the YAML file. For example, LLMs can successfully specify an AppArmor profile in a Helm chart, but defining the corresponding profile is a completely different task that requires further and more complex interaction with the model, if possible in the first place. Finally, in terms of LLM refactorings correctness (RQ3), through manual validation we found that such models have very low performances in refactoring cloud misconfigurations. This suggests that LLMs can still be used but require manual validation and interpretation, to avoid changing and removing irrelevant lines; also, extracting the LLM’s answer snippet and embed it into a chart is a complex task to automate, and should better be done manually. A common definition of when a configuration is a misconfiguration, to avoid tool’s inconsistencies, and the use the LLMs also in the detection phase can lay the base for future work.

### Registered Report

This paper is the result of executing the method proposed in a previous peer-reviewed registered report (Minna et al. 2024), presented at the Mining Software Repository 2024 conference. The registered report (RR) track follows a two step process, where the authors first submit a plan of the study they intend to conduct, and then, upon peer-review acceptance, they conduct the study, generating the results and collecting the data. In this paper, we executed the method proposed in the previous peer-reviewed registered report, along with some relevant improvements, such as using more LLMs, and presenting the final results.

The rest of this paper is organized as follows. In the next section §2, we provide some background and related work about Helm Charts security and configuration repair. In §3, we present our research questions, and in §4 we present our execution plan to answer such questions. Then we answer each research question in a separate section, namely, §5, §6, and §7. Finally, in §8 we discuss our findings, concluding with the threats to validity §9.

## Background and Related Work

This section presents the terminology, background, and related work on the Helm package manager, Helm charts, and tools that can analyze such charts.

### Terminology

We hereby provide some terminology about cloud configurations useful to understand the rest of this paper.

A cloud **configuration** is a specification in a Domain Specific Language (DSL) that describes how a cloud resource has to be deployed. For example, a YAML file of a Helm chart specifying the amount of memory and CPU for a certain container.

We consider such a configuration correct if it can be deployed on a platform without any error; for example, a Helm chart without syntax error accepted by a Kubernetes cluster.

A **safe configuration** is a correct configuration that adheres to best practices, e.g., the K8s CIS Benchmark and Helm chart analyzer tool policies.

Instead, an **unsafe configuration** is a correct configuration that does not adhere to best practices, e.g., the K8s CIS Benchmark and Helm chart analyzer tool policies. Note that some applications may require unsafe configurations (e.g., not read-only filesystem and additional Linux capabilities) to function properly, e.g., intercepting network packets or making changes to files. Unsafe configurations should still adhere to the principle of least privilege. For example, a container with granular permissions (e.g., a subset of capabilities) is more secure than a privileged container (i.e., with all capabilities).

Based on the previous definitions, we provide a definition for a misconfiguration.

A **misconfiguration** is a configuration that is wrong, i.e., does not comply with a platform supported DSL’s rules, and/or unsafe, i.e., that does not adhere to best practices or has more permissions than necessary to perform a certain task.

### Kubernetes Background

We hereby provide a brief overview of a K8s cluster and its components.

An application running on K8s is deployed within a *cluster*, that is a set of either virtual or physical machines. In every cluster there is (at least) one master node and several worker nodes; master nodes have the task of managing all components and keeping the application at the desired state, whereas the worker nodes are the power units. Pods, Deployments, and StatefulSet are examples of resources that could be deployed on a K8s cluster. A pod is the smallest deployable object in a cluster, containing at least one container; usually, pods are used to run testing containers and helper processes. A Deployment is a higher abstraction of Pods that allows to manage a specified number of replicas of a Pod, handling the creation, scaling, and updates of those; usually, Deployments are used for applications that require multiple replicas for load balancing and rollouts and rollbacks. A StatefulSet is a specialized resource that manages the Deployment and scaling of a set of Pods, ensuring, at every time, that each Pod has a unique and persistent storage; usually, StatefulSets are used for applications that need to maintain states, such as databases. Usually, an application is composed of a combination of such resources, along with other resources, such as scripts, configuration setups, and secrets. For a detailed explanation of each cluster components, we redirect the reader to the official K8s documentation (Kubernetes 2024).

### Infrastructure as Code and Helm Charts

Infrastructure as Code (IaC) enables provisioning, managing, and configuring infrastructure resources (such as virtual machines and containers) using configuration files, rather than manual processes. This allows for easy versioning, sharing, and scaling. Previous work has already investigated the security of IaC scripts, for example, to identify defects and privacy violations (Rahman 2018) and to predict and identify defects in open-source project commits (Rahman et al. 2018). Rahman et al. (2019) evaluated 1, 726 IaC scripts to identify the presence of seven security smells — weak code patterns that can lead to vulnerabilities, e.g., suspicious comments and weak cryptographic algorithms, the frequency, and mitigation lifetime. The same authors found 9, 175 hard-coded passwords in IaC scripts (Rahman and Williams 2021).

Kubernetes (K8s) is a container orchestration engine that enables the efficient management, deployment, and scaling of hundreds of containers using YAML configuration files. Helm is a package manager for K8s that allows to define, manage, share, version, and deploy applications as a set of directories and YAML files, called charts. Artifact Hub (The Linux Foundation 2024) is a repository where developers and organizations can upload, share, and download Helm chart artifacts; in other words, it is the equivalent of Docker Hub for container images, but for Helm Charts.

### Security Issues in Helm Charts

As pointed out by previous work, K8s configuration files or manifests contain misconfigurations and security defects. For example, Bose et al. (2021) and Rahman et al. (2024) investigated the type and frequency of security defects (e.g., buffer overflow and denial of service) that appear in K8s manifest from open-source projects. Islam Shamim et al. (2020) surveyed 104 grey literate source and suggested 11 security best practices, such as CPU and memory request limits, network policies, and avoiding the use of default namespaces. Finally, Blaise and Rebecchi (2022) used topological graphs to represent Helm Charts and scored each deployment resource based on best practices, container image vulnerabilities, and potential attack paths.

Besides academic contributions, there also exist several tools that can detect misconfigurations, security risks, and best-practice violations in such charts before deployment. Examples of such tools are Checkov, Datree, KICS, and Terrascan. To the best of our knowledge, a study investigating the characteristics and performance of such tools is still missing.

### Misconfiguration Repair

Automatic Program Repair (APR) techniques aim to automatically identify patches for a given error or bug, minimizing human intervention, which is lately receiving a lot of attention. Pinconschi et al. (2022) proposed MAESTRO, an APR pipeline to test several vulnerability benchmarks (e.g., Defects4J and CB-repair) and APR techniques (e.g., GenProg and MUT-APR) for C and Java programs; they evaluated the pipeline on ten projects, evaluating correctness and time overhead. Tjiong et al. (2022) proposed F1X, an APR tool to fix security vulnerabilities found by OSS-Fuzz, a fuzzing framework to find vulnerabilities in open-source projects; they evaluated the tool on 240 vulnerabilities found by OSS-Fuzz in five C open-source projects, measuring correctness compared to OSS-Fuzz suggestions. Fu et al. (2022) proposed VulRepair, a T5-based (a Transformer-based Neural Machine Translation — NMT) vulnerability repair approach; they evaluated the approach on more than 8, 000 vulnerability fixes from 1, 754 open-source projects, measuring accuracy and fix prediction.

Large language models (LLMs) are a type of artificial intelligence (AI) based on deep learning algorithms, trained on a massive amount of text data, that can perform several natural language processing (NLP) tasks. For example, they can generate new text, translate, understand contexts, and answer questions; in other words, they output the most likely next word based on the given input. Google Gemini and GPT are two common examples of LLMs.

LLMs have already been investigated in previous work, discussing the advantages and disadvantages of usages for software engineering practices (Fan et al. 2023), presenting a prompt patterns catalog for software engineering tasks (White et al. 2023), and evaluating the code generation capabilities of both ChatGPT and Copilot (Hansson and Ellreus 2023). Biswas (2023) discussed ChatGPT software programming capabilities; among other results, they concluded that ChatGPT can perform code completion, correction, and refactoring while providing users with explanations. Sokolowski and Salvaneschi (2023) proposed an automated testing tool to fuzz Pulumi TypeScript IaC programs for reliability, and testing configurations for termination, correctness, and compliance. However, besides the detection and refactoring advantages of using LLMs, there is also the concern of recommending or generating insecure code, as shown by Pearce et al. (2022). Similar concerns have also been documented in the grey literature, highlighting both the increased adoption as well as the generation of insecure code (Snyk 2024). Therefore, the manual validation allowed us to evaluate the reliability of using LLMs for detecting and refactoring misconfigurations.

To the best of our knowledge, prior research did not propose or investigate the use of APR or LLMs to detect and repair Helm charts.

## Research Questions

The goal of this research is threefold: investigate what misconfigurations are reported by existing tools, evaluate LLMs’ refactoring capabilities in removing misconfigurations, and finally, manually measure the robustness of both tools and LLMs in terms of misconfigurations true positives and false negatives.

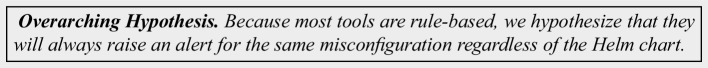



For example, a privileged container will always be detected, despite whether it is defined within a StatefulSet, a Deployment, or a Pod. We plan to confirm this hypothesis by analyzing all (thousands of) charts available on Artifact Hub.

Specifically, we first aim to systematically evaluate a set of open-source analyzer tools on all Helm charts to measure how many misconfigurations are reported, of what type, whether there are certain misconfigurations reported by only a subset of tools (i.e., unique policies), and which is the tool with the best coverage (i.e., that finds most misconfigurations).

- **RQ1.** *What are the misconfigurations reported in Helm charts on Artifact Hub?*

Answering this question allowed us to measure each tool’s performance and inconsistencies on the same charts, which highlights the limitations of current solutions, and identify the most common misconfigurations, which can be a useful starting point for practitioners to secure their running applications.

Second, automatically suggesting how to remove misconfigurations is still an open question, as it may depend on the domain of the application, and a fixed set of mitigations may not be an appropriate solution. Therefore, it is interesting to investigate whether LLMs can be used to refactor charts by removing such misconfigurations.

- **RQ2.** *Can LLMs refactor Helm charts and remove misconfigurations?*

To this aim, we queried LLMs to refactor the chart for each misconfiguration detected in the previous step. However, LLMs can hallucinate, i.e., generate wrong and inconsistent results, because, for example, of flawed training data sources (Huang et al. 2023). Therefore, because of such hallucination problems, LLMs can refactor a chart with both secure and insecure configurations. For example, it might be that mitigations are correct only for very simple modifications (e.g. replacing an empty memory requirement with memory requirements with a constant value), but more complex suggestions might escape detection by the toolchain. Therefore, we also wanted to evaluate the mitigations implemented by LLMs.

- **RQ3.** *Are there false positives in the analyzer tool results and hallucinations in LLM refactoring?*

We answered this question in two steps. First, we run the tools again on the chart refactored by an LLM and compare the output with the results of RQ1. In other words, we measured how many misconfigurations implemented by the LLM satisfy each tool policy. Furthermore, because LLM can hallucinate, or analyzer tools can produce false positive results, we performed a manual validation on a subset of charts to check whether misconfigurations are actually present or not and whether the implemented mitigations are logically correct.

For each RQ, we collected several metrics and evaluate each tool individually using statistical tests. For RQ3, we also performed a manual validation on a sub-sample of the results to provide the Agresti-Coull-Wilson interval for the reliability of our estimates. This interval allowed us to extrapolate the result of our manual validation on the dataset subsample and extend the conclusion to the all dataset.

## Execution Plan

**Fig. 1:**
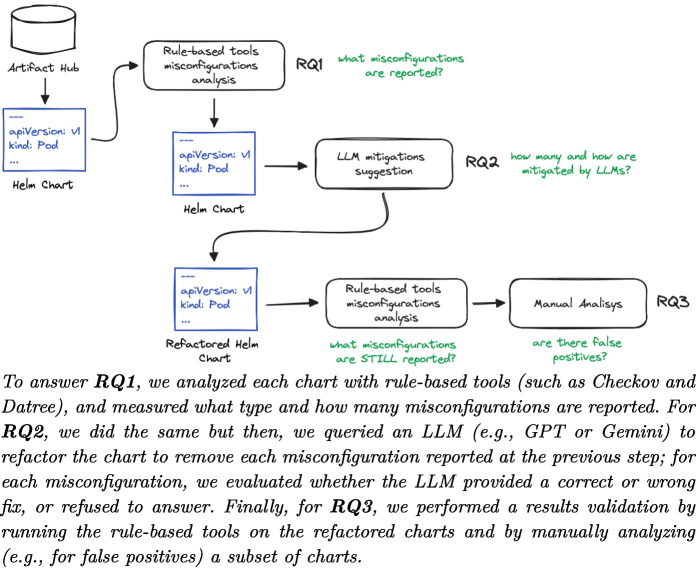
The execution plan of our experiment.*Note.* To answer **RQ1**, we analyzed each chart with rule-based tools (such as Checkov and Datree), and measured what type and how many misconfigurations are reported. For **RQ2**, we did the same but then, we queried an LLM (e.g., GPT or Gemini) to refactor the chart to remove each misconfiguration reported at the previous step; for each misconfiguration, we evaluated whether the LLM provided a correct or wrong fix, or refused to answer. Finally, for **RQ3**, we performed a results validation by running the rule-based tools on the refactored charts and by manually analyzing (e.g., for false positives) a subset of charts

In this section, we outline the approach used to answering our research questions. Figure 1 summarizes the execution plan, where each step is further explained in the following subsections.

### Helm Charts Full Dataset

**Fig. 2:**
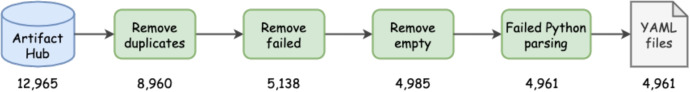
The steps to download and generate the Artifact Hub chart’s templates

We used the Artifact Hub API /helm-exported command^2^ to download the latest version available of all charts listed in the repository. Figure 2 shows all the steps, along with the corresponding number of charts, that we followed to download and generate the local YAML template file for each chart available.

In total, we downloaded 12, 965 charts; after removing duplicates, 8, 960 charts remained. Using the helm template command, we correctly generated 5, 138 chart YAML template files. For 3, 822 charts, the template command failed for different reasons, for example, “ A valid.Values.config.secureAPIToken is required” and “Operator [cloudnative-pg] has to be installed first”; for this experiment, we only considered templates that could be generated without any manual pre-setup or customization. Instead, for 153 charts, the template command generated empty YAML files, that we also discarded. Finally, for 24 templates, Python failed to parse the YAML file, for example, because of “yaml.scanner.ScannerError: while scanning for the next token found character \t that cannot start any token”. In the end, we correctly generated 4, 985 YAML template files.

Table 1 provides the list of the ten most popular Helm Charts available on Artifact Hub. For each chart, it provides the name, repository (to uniquely identify a chart), number of stars (used to rate the chart’s popularity), the number of YAML chart template file lines, and the number of containers.

**Table 1:** Top ten most popular Helm charts in Artifact Hub

Name	Repository	Stars	Lines	#containers
kube-prometheus-stack	prometheus-community	572	44, 331	11
ingress-nginx	ingress-nginx	465	725	3
cert-manager	cert-manager	446	1, 313	4
argo-cd	argo	325	18, 539	9
prometheus	prometheus-community	297	1, 278	6
redis	bitnami	269	645	2
grafana	grafana	258	332	2
kubernetes-dashboard	k8s-dashboard	220	532	1
postgresql	bitnami	218	225	1
traefik	traefik	175	265	1

**Fig. 3:**
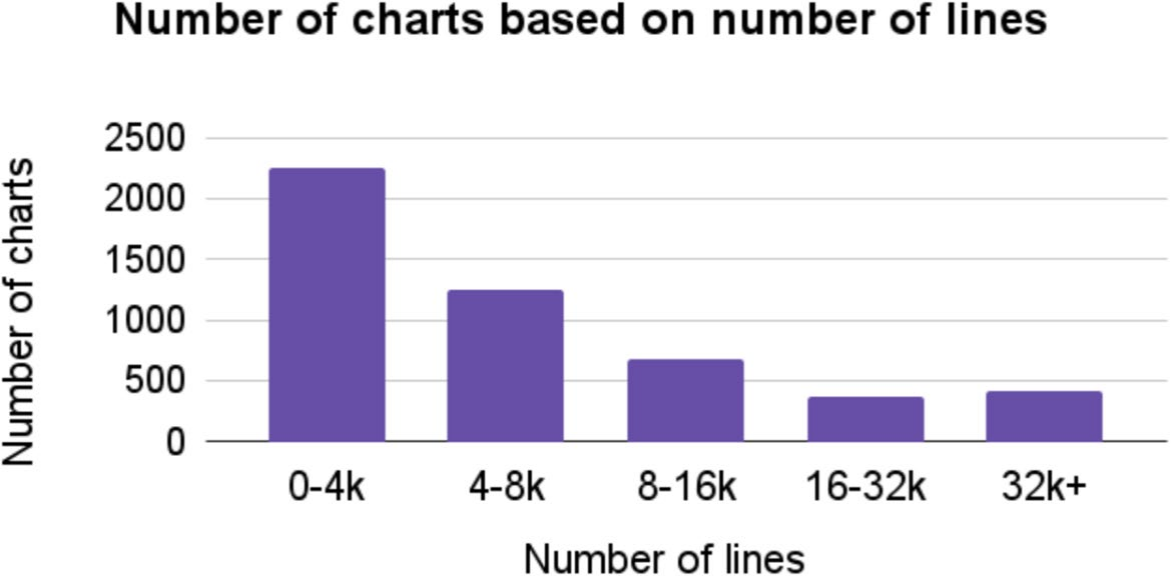
The distributions of chart templates based on the number of lines

Figure 3 shows the distributions of chart templates based on the number of lines.

Table 2 shows, instead, some statistics about the chart templates, specifically, the min, max, mean, median, standard deviation (SD), first quartile, and third quartile of the number of lines and number of containers.

**Table 2:** General statistics about the number of lines and containers in the chart templates

	Min	1st quart.	Median	3rd quart.	Max	Mean	SD
Cont.	0	1	1	2	56	2.21	3.31
Lines	8	106	384	2,786.87	53,691	701.44	193

### Helm Charts Smaller Dataset

We created a smaller Helm charts dataset to evaluate two more LLMs by reducing the number of charts and thus queries (saving both on costs and time). To select the charts, we counted the number of semicolons in each template; we used the number of semicolons as a metric to measure how *complex* was each chart template. Indeed, because in YAML syntax each semicolon corresponds to an assignment of a value to a key, the more semicolons there are, the more objects there are in the template. Using this metric, we selected a subset of all chart templates while making the LLMs evaluate all different complexity levels.

Table 3 shows some statistics about the number of semicolons across the all chart dataset, specifically, the min, max, mean, median, standard deviation (SD), first quartile, and third quartile of the number of lines and the number of containers.

**Table 3:** General statistics about the number of semicolons across the all chart dataset

	Min	1st quart.	Median	3rd quart.	Max	Mean	SD
Semic.	7	98	168	326	100,611	601.89	2,716.81

To select the charts, we used ranges of 100 semicolons each; thus, in total, we have 1, 006 ranges (i.e., 0-100, 100-200, 200-300, etc.). For each range where there is at least one chart, we selected the first one to appear. Table 4 shows the number of charts for the first ten ranges of the number of semicolons.

**Table 4:** The number of charts for the first ten ranges of the number of semicolons

Range	# Charts
0 - 100	1278
100 - 200	1525
200 - 300	779
300 - 400	398
400 - 500	204
500 - 600	133
600 - 700	83
700 - 800	61
800 - 900	64
900 - 1000	38

As you can see, of the 4, 961 total number of templates, most of them (4, 563) belong to the first ten ranges of the number of semicolons. Indeed, because some ranges do not have any chart belonging to them, we finally have **126** charts (e.g., there are no charts with the number of semicolons between 4000 and 4100).

### Analyzer Tools

We retrieved open-source Helm Charts and YAML file analyzers either from the CNCF projects list or from GitHub, listed in Table 5. We used these tools to analyze each chart and answer our research questions.

**Table 5:** Open-source Helm Charts and YAML analyzers

Tool	Company	CNCF	Policies	Fix	Link
Checkov	BridgeCrew	✓	38		PrismaCloud (2024)
Datree	Datree	✓	38		Datree (2024)
KICS	Checkmarx	✓	31		Checkmarx (2024)
Kubelinter	StackRox		51		StackRox (2024)
Kubeaudit	Shopify		14	✓	Shopify (2024)
Kubescape	ARMO	✓	50		Armo (2024)
Terrascan	Tenable	✓	33		Tenable (2024)

All tools are open-source static-time analyzers that can check Helm Charts and K8s manifests. We selected the tools that are part of the CNCF as “graduated” or “incubating” projects. In addition, we included Kubelinter and Kubeaudit, retrieved from GitHub, to further increase the sample of existing tools (see Table 5). Each tool supports different policies to check for misconfigurations in K8s manifests. Example policies include ensuring that each container has a configured memory limit, that container CPU requests are not equal to its limits, minimizing the admission of containers with the SYS_ADMIN capability, and ensuring that each container image has a pinned (tag) version. Also, all tools allow defining custom policies to ignore specific resources, specify whether to scan a Helm Chart folder, a set, or a single YAML file, and provide different output formats from the console to the JSON format. We did not consider PodSecurityPolicy, because it was officially deprecated in K8s v1.21, and infrastructure policies (e.g., "*Ensure that the–kubelet-https argument is set to true*") because it is not possible to check them by analyzing Helm Charts.

### Misconfiguration Mapping

Because every chart analyzer tool has a different type and definition of policies, to compare the results of such tools, we need to map each tool’s policy and output format to a common framework. In fact, even for the same CIS Benchmark recommendations, e.g., ”*Minimize the admission of containers with allowPrivilegeEscalation*“ (5.2.5), tools can have different policies checking for this property, each policy can be implemented differently, and the alert (tools’s output format) may be syntactically different. Such differences make it not trivial to parse each tool’s output and require manual effort even for something as easy as counting the number of misconfigurations found.

The technical challenge of this step is examining the programming function(s) implementing the same policy checks across different tools to determine whether the implementation of two policies is equivalent or not, even if the alert is syntactically the same. This significant manual effort resulted in building up a framework that can be used to automatically analyze and compare different tools. For each policy, to determine equivalence or not, we strictly followed a three-step process and checked the actual implementation, i.e., which YAML keys and values are evaluated.

- TC_0.1. *Map policy to the CIS Benchmark recommendations*. First, we check whether the policy is related to one of the CIS recommendations or not; if yes, then we map the policy to the standard ID for that recommendation. Some tools have the same policies checking for different configurations, in which case, we report the same policy ID for each standard ID.
- TC_0.2. *Map unique policy or alternative policies in common with a subset of tools*. If the policy is not related to the CIS recommendations, we evaluate what properties (i.e., YAML keys and values) are related to; if the same properties are also checked by another tool’s policy, we map the two policies to the same standard ID as equivalent, otherwise is a unique policy.
- TC_0.3. *Classify policy as related to functionality or best practice*. Finally, we categorize each policy as either related to configuration functionality or best practice. Specifically, a configuration is related to functionality if it can break the application, otherwise, we consider it best practice.

Table 6 shows, for each tool, the number of policies supported by only that tool (column 1), the number of policies supported by that tool and another tool (column 2), the number of policies supported by that tool and other two tools (column 3), and so on, along with the total number of policies. For each tool, the total number of policies is based on the number of default policies from that tool that were found at least once in the chart dataset, along with how many of these were related to functionalities (Func.) or best practices (BP).

**Table 6:** Tools comparison between the supported policies

Tool	1	2	3	4	5	6	All	Total	Func.	BP
Checkov	1	1	2	2	9	6	9	30	20	10
Datree	2	1	4	1	9	7	9	29	16	13
KICS	8	1	5	2	9	7	9	41	19	22
Kubeaudit	0	0	2	0	4	4	9	19	13	6
Kubelinter	1	1	1	0	2	6	9	20	17	3
Kubescape	1	1	2	1	6	7	9	27	17	10
Terrascan	0	1	2	3	6	4	9	25	16	9

For each tool (row), we show the number of unique policies (column 1), the number of policies in common with another tool (column 2), and so on, with the total number of policies, found at least once in the dataset. For example, Kubeaudit and Terrascan have at least one policy in common with another tool, there are only nine policies in common between all tools, and there are more policies related to functionalities than best practices

From this table, we can see that KICS is the tool with the most unique policies, thus checking more different configurations by default, Kubeaudit, and Terrascan have at least one policy in common with another tool, and that there are only nine policies in common between all tools; also, there are always more policies related to functionalities than best practices.

We hereby describe the methodology by which we decide whether to add a new reported policy by a tool or not to the mapping.

1. If the policy is already in the mapping, add the tool’s alert ID.
2. If the policy occurs in multiple charts (more than 1% of the charts), then we map it, otherwise not.
3. If there is one chart with several tools and unmapped misconfigurations, we map it (because we don’t know if they are equivalent or not).

Using this methodology, we assume that whatever policy is not mapped, is not making a significant difference (e.g., in counting the most common misconfigurations); therefore, we also query LLMs to mitigate misconfigurations that have been mapped.

### LLM API Engineering

Compared to the original registered report, we used four LLMs, instead of two, to query for possible mitigations, specifically, the original models, GPT-4o-mini-2024-07-18 (OpenAI 2024), Google Gemini 1.5 (Google 2024), and two more, specifically, Anthropic Claude-3-sonnet (Anthropic 2024), and Meta Llama3-70b (Meta 2024). The reason for this change in using and comparing two more LLMs was due to the feedback received during the presentation of the original registered report at the MSR’s 24 conference, about the costs and time needed to replicate the study. In fact, using only proprietary models would increase the upfront cost that researchers have to pay for replicating the results, making it less accessible. At this time of submission, the Llama model has been released as open source, the Claude model is available on several platforms for different costs (e.g., AWS and Hugging Face), and GPT API costs are significantly lower compared to before. We truly believe that using two more models provided more insightful results and made applicability more accessible.

To interact with each model, we used the available Python API libraries, available for each model (e.g., Python SDK for the Gemini API^3^). We used the models out of the box, without any training or further customization. Algorithm 1 shows the Python function pseudocode to query a model using the APIs and a list of queries.

**Algorithm 1:**
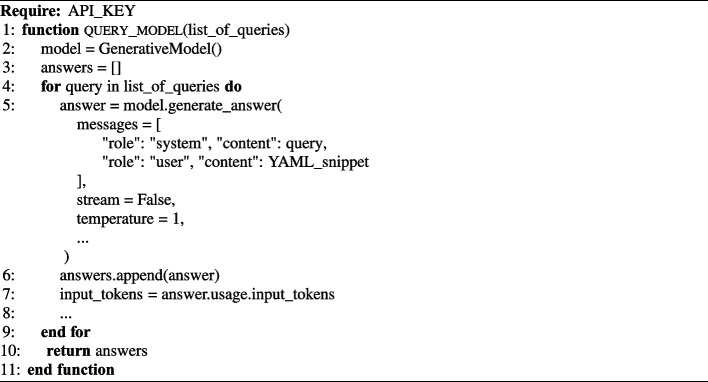
Python pseudocode to query a model using the APIs and a list of queries.

The QUERY_MODEL function provides a generalization of the Python function that is available to invoke each model. The arguments of the model.generate_answer() functions are usually the same for all the models, for example, the list of messages, temperature, max_output_tokens, etc.; for our experiment, besides the provided query, we used the default settings. Also, the function is wrapped around a try and except block to catch any exception from the model (e.g., google.api_core.exceptions.InternalServer Error).

Regarding the messages, for each query, we first specify the context (e.g., You are a software engineer working on a Kubernetes project...) along the failed policy text description (e.g., memory limits should be defined for each container), and then we provide the YAML_snippet against which the policy failed; further details of each query format are discussed in §4.6.

It is important to highlight the fact that there is no context sharing or remembering from the LLM across the queries. In fact, LLM APIs, by default, are stateless, i.e., they have no memory of previous conversations. To make the model consider previous input and answers, the user must specify these latter, usually in the messages argument of the API model function. Chatbots like ChatGPT provide a wrapper around this API function, by appending the model answer and the new user input to continue the conversation taking into account the previous interactions. For our experiment, we only provided a single query as the argument, so that each refactoring is independent of the others.

### LLM Query Engineering

At the time of the experiment, the following were the corresponding LLM input and output token limits: 128, 000 input tokens and 16, 384 output tokens for GPT-4o-mini-2024-07-18, 1, 048, 576 input tokens and 8, 192 output tokens for Google Gemini 1.5, 40, 000 input tokens and 8, 000 output tokens for Anthropic Claude-3-sonnet, and 8, 196 input tokens and 2, 048*tokens* output tokens for Meta Llama3-70b.

Because of the limited input size that LLM chatbots can parse, and because Helm chart templates can contain up to thousands of lines, we can not provide the whole chart as input. However, misconfigurations affect either one line or a single YAML object, which is reported by existing tools along with the violated policy.

Therefore, to answer both RQ2 and RQ3, we queried LLM chatbots to refactor snippets of Helm charts to remove the misconfigurations that were detected in the previous step.

The code snippet was extracted from the whole chart by using the misconfiguration location provided by each tool, and a Python script. In fact, every tool, for each misconfigurations, reports the affected object (e.g., Pod, or Deployment), deployment namespace, and name; the combination of these three information allows to uniquly locate the affected resource within the all YAML file.

Specifically, each query included i) a K8s resource, ii) a misconfiguration, and iii) the resource YAML code; instead, the second part of the query specified the format of the chatbot output for further processing.

For example, for a Deployment object and the “Prevent containers from escalating privileges” policy^4^, the query was the following:

*Refactor the following Deployment K8s resource to prevent containers from escalating privileges. Output only the refactored YAML file. [YAML code...]*

**Example on Listing 1 and Datree tool.** Listing 1 shows an example of a busybox Pod without resources memory requests defined. Datree will raise an alert when analyzing this resource, highlighting that memory requests should be defined.
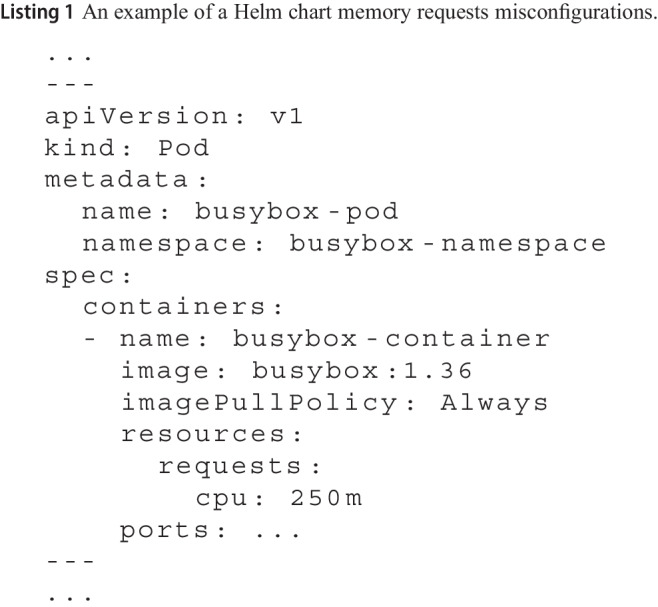


To refactor this resource, we can give as input the K8s resource, i.e., Busybox pod, and the violated policy, i.e., “Ensure each container has a configured memory request”^5^ to LLM chatbots, along with the Pod YAML code shown in Listing 1, as follows:

*Refactor the following Pod K8s resource to ensure each container has a configured memory request. Output only the refactored YAML file.*

Listing 2 shows the refactored Pod by Google Gemini, using the previous query. When this refactored chart is checked by Datree again, no memory limit alert is raised, confirming that the generated suggestion satisfies the memory requests policy.
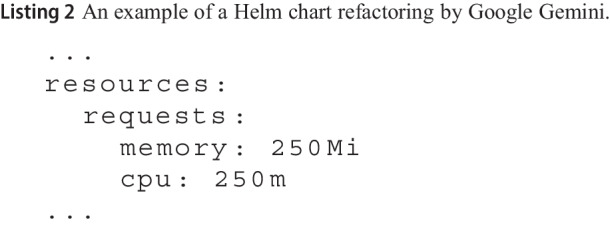


Because every tool, for each failed policy, outputs a text description of the corresponding policy (e.g., ”*Ensure each container has a configured memory request*“), we used this description to build the query for the LLM. This allowed us to craft all the queries from each tool output automatically; also, because for equivalent policies of different tools, the description can be slightly different, this allowed us to evaluate whether a different description yields better mitigation or not, for the same misconfiguration.

### LLMs Interaction Process

We implemented the proposed pipeline to automatically find misconfigurations and use LLMs for remediation using a Python program and each LLM API. Specifically, at the beginning, one of the tools analyzed a chart and output the results in YAML format; for each tool and chart, the result YAML file includes a unique identifier to the failed policy (e.g., container without memory limits), and the resource to which the misconfigurations refers to (i.e., resource type, name, and namespace). Therefore, first, the Python program parsed the tool analyzer output, and for each reported misconfiguration, it located and extracted the YAML snippet of the affected resource from the chart. Using the policy text description and the resource YAML snippet, the program then queried the LLM, asking to refactor the resource to remove the misconfigurations. While querying the LLM, the program also asked to only output the refactored YAML file, such that it can be locally saved and compared to the original YAML snippet. In the end, after saving the LLM output, the program compared with a diff the original YAML snippet with the LLM output YAML snippet to check whether mitigation was implemented or not. For the charts and misconfigurations refactored by the LLM, we ran the analyzer tools again and performed a manual analysis on a subset of charts to check whether the mitigations satisfy the tool policies and are logically correct (e.g., memory is not equal to 0).

## RQ1: Reported Misconfigurations

We run seven chart analyzer tools across 4, 961 chart templates. We now report several statistics and findings about the tools, e.g., the average number of misconfigurations found, and the most common misconfigurations. Table 7 shows, for each tool, the number of charts successfully analyzed with at least one misconfiguration found (“1+ Alerts”), the number of charts that the tool failed to analyze (“Failed”), and the number of charts with no misconfigurations found (“No Alerts”).

**Table 7:** Statistics about the number of charts analyzed by tool

	1+ Alerts	Failed	No Alerts
Checkov	4,434	1	526
Datree	3,220	**1,538**	203
KICS	4,563	0	398
Kubeaudit	4,248	260	453
Kubelinter	4,379	0	**582**
Kubescape	**4,766**	5	190
Terrascan	4,288	165	508

Kubelinter is the tool that did not raise any alert in most charts likely because is the tool with the smallest set of policies available by default; because of this, and because it also has one unique policy (as shown in Table 6), it may be worth using other tools.

Datree is the tool that failed to analyze most charts likely because it checks for the syntax, and if the YAML syntax is wrong, it will not evaluate any policy. Thus Datree can be a good choice to check also for syntax, before misconfigurations, without using another tool.

Table 8 shows, for each tool, the statistics about the number of alerts raised by each tool on a single chart, namely, min, median, max, mean, and standard deviation (SD) of number of alerts per chart.

**Table 8:** Statistics about the number of alerts per chart by tools

	Min	1st quart.	Median	3rd quart.	Max	Mean	SD
Checkov	0	15	20	37	786	33	44
Datree	0	0	6	11	202	8	12
KICS	0	18	24	44	1097	46	68
Kubeaudit	0	6	8	14	325	13	21
Kubelinter	0	3	6	13	300	12	20
Kubescape	0	6	10	19	682	18	28
Terrascan	0	7	11	19	384	17	24

KICS is the tool with the most policies available by default, thus, it is likely the tool that finds the most misconfigurations per chart, as reflected by the mean of alerts raised for each chart.

To compare the type and number of misconfigurations, we created a policy mapping, introduced in §4.4, which allows a fair comparison of the tool’s performance. For example, we mapped the policy “ensure each container has configured memory limits” to ID check_1 and the corresponding policy from each tool, if supported, e.g., CKV_K8S_12 for Checkov, 229588ef-... for KICS and AC_K8S_0099 for Terrascan.

Also, for further comparison, we counted the occurrences of policies based on the CIS Benchmark clusters. For this experiment, we only considered the clusters relevant to Helm charts and configuration files, i.e., the fifth paragraph about Policies; we ignored other recommendations from paragraphs out of scope for the charts, such as the third (Control Plane Configuration) and the fourth (Worker Nodes). Specifically, we considered six clusters of policies from the benchmark, namely, *RBAC and Service Accounts* (benchmark section 5.1), *Pod Security Policies* (benchmark section 5.2), *Network Policies and CNI* (benchmark section 5.3), *Secrets Management (benchmark section 5.4)*, *Extensible Admission Control (benchmark section 5.5)*, and *General Policies (benchmark section 5.6)*. Table 21 in the Appendix shows the mapping between each policy ID and the corresponding CIS Benchmark Cluster.

Table 9 shows the top ten failed policies across all charts dataset, along with the number of tools that support the policy, a brief description, the percentage of charts in which it failed, and the corresponding CIS Benchmark cluster.

**Table 9:** Top ten mapped policies across all charts

ID	# Tools	Description	% Charts	CIS Cluster
26	5	Using default namespace	90%	General Policies
32	3	Missing AppArmor profile	90%	-
31	5	Missing SecComp profile	89%	General Policies
48	1	Missing LimitRange	88%	-
49	1	Missing ResourceQuota	88%	-
40	4	Pod without NetworkPolicy	88%	Network Policies and CNI
13	5	Using privileged UIDs	87%	-
22	7	Missing privilege escalation prevention	86%	Pod Security Policy
27	7	Missing read-only fs	83%	-
34	5	Minimize capabilities	82%	Pod Security Policy

Across all alerts, the policies belonging to the Pod Security Policy CIS Benchmark cluster (e.g., Missing privilege escalation prevention— policy 22, and minimize the assigned capabilities — policy 34 in Table 9) are the most common (133, 325 occurrences), followed by policies belonging to General Policies (114, 263 occurrences), RBAC and Service Accounts (47, 195 occurrences), Network Policies and CNI (24, 966 occurrences), and finally Secrets Management (10, 565 occurrences) clusters.

Likely due to the complexity of being too fine-grained, PodSecurityPolicy was deprecated in Kubernetes v1.21, and removed from Kubernetes in v1.25; the alternative is to define Pod Security Standard at the namespace level or use an external 3rd party admission plugin.

By creating a namespace object, it is possible to specify the Pod Security Standards by defining the corresponding labels. Specifically, there are three different policies, namely, privileged (everything allowed), baseline (minimally restricted), and restricted (following all best practices), and three possible violation actions, namely, enforce (reject configuration), audit (create a log), and warn (trigger a warning). Similarly, to use an admission controller, the user can create an AdmissionConfiguration object, with the corresponding permissions.

We also claim that RBAC, network, and secrets-related policies are less common because external components usually manage them; for example, network policies are usually created on the fly by the CNI plugin after monitoring the cluster network traffic, and secrets are stored outside K8s, e.g., by the Hashicorp Vault. The static tools we tested in this experiment can not evaluate the configuration and information handled by external components.

Even though Pod Security Standards can be defined as objects within the YAML configuration files, like the PodSecurityPolicy, to the best of our knowledge, none of the tested tools have policies (available by default) that check for their definition of Pod. This is a significant limitation of the tools that should be updated to follow the new K8s versions, to avoid generating misleading results.

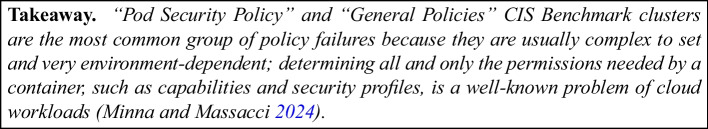



## RQ2: LLMs Refactoring

### Query Datasets

#### Standard Dataset

In total, all tools raised 698, 222 alerts across all templates, which would correspond to as many LLM queries. To reduce costs to replicate our study, we retained only the first occurrence of each alert for each chart; for example, if there are two missing memory request alerts in the same chart referring to two different resources, we only consider the first alert. This allowed us to lower the total number of queries to 297, 424. Additionally, because some tools do not have unique policies, specifically, Kubeaudit and Terrascan, we assume that such tools do not raise any alert that at least another tool would not raise, and therefore removed the alerts of such tools as well. This allowed us to further lower the number of queries to 229, 183. On this dataset, we used two LLMs, namely, GPT-4o-mini-2024-07-18 from OpenAI, and Claude-3-sonnet from Anthropic.

#### Smaller Dataset

As explained in §4.2, we counted the number of semicolons in each chart and used it as a metric to evaluate the complexity of each chart, finally selecting one chart for each number of semicolons range. This led us to a final number of 126 charts; by only considering the alerts related to these charts in the standard dataset, we finally obtained 7, 206 queries. On this dataset, we used the other two LLMs, namely, Gemini 1.5 (Google 2024) from Google, and Llama3-70b from Meta.

### Query Evaluation

For both datasets, each query was composed of the context description along with the K8s resource type, the tool’s policy text description of the misconfiguration, and the corresponding resource YAML snippet, as described in § 4.6. Table 10 shows, for each LLM, the number of total queries performed, how many were successful, and how many failed.

**Table 10:** Performance of LLMs in terms of answering the queries

		Claude	Gemini	GPT	Llama
Total Queries		229,183	7,206	229,183	7,206
Generated Answer		99%	89%	99%	89%
Failed to parse YAML		1%	1%	1%	10%
Failed to generate answer		$<1$%	10%	0	$<1$%
Total Failed Queries		1%	11%	1%	11%

In several cases, LLMs generated an answer with a wrong YAML syntax in one or more lines; in such cases, the Python library that we used to parse the answer raised one or more exceptions. We counted such cases where a Python exception was raised due to a wrong YAML syntax in the “Failed to parse YAML answers” metric and not further analyzed that answer.

Regarding the other failed answers, the error may not be related to the model, but to the network connections, LLM APIs, other Python libraries, etc., thus we count them as general errors.

Table 11 shows, for each LLM, some statistics about input and output tokens, and the number of added, changed, and removed lines. We only counted input and output tokens when the LLM model provided (syntactically correct) answers.

**Table 11:** LLM answer statistics about tokens and changed lines

		Claude	Gemini	GPT	Llama
Input Tokens	**Min**	54	43	55	62
	**1st quart.**	203	199	172	202
	**Median**	351	367	289	343
	**3rd quart.**	648	730	521	640
	**Max**	109,218	87,476	85,734	7,945
	**Mean**	513	588	422	473
	**SD**	789	2,094	740	400
Output Tokens	**Min**	22	1	15	1
	**1st quart.**	189	172	170	194
	**Median**	318	390	288	352
	**3rd quart.**	549	699	473	512
	**Max**	4,096	8,192	9,054	512
	**Mean**	438	511	382	334
	**SD**	395	556	344	162
Added lines	**Min**	0	0	0	0
	**1st quart.**	0	0	3	0
	**Median**	2	3	11	6
	**3rd quart.**	9	15	19	18
	**Max**	384	526	179	96
	**Mean**	6	8	12	10
	**SD**	11	13	9	11
Changed lines	**Min**	0	0	0	0
	**1st quart.**	0	0	0	0
	**Median**	0	0	0	0
	**3rd quart.**	0	0	1	1
	**Max**	19	9	10	7
	**Mean**	0	0	0	0
	**SD**	0	1	1	1
Removed lines	**Min**	0	0	0	0
	**1st quart.**	0	0	0	0
	**Median**	0	0	0	0
	**3rd quart.**	0	0	0	0
	**Max**	15,951	14,707	15,718	581
	**Mean**	1	7	2	9
	**SD**	36	203	43	27

Every LLM uses a different algorithm to convert the input text to tokens; therefore, for the same input, LLMs can generate different numbers of tokens. By looking at the token statistics, we can conclude that a higher number of tokens (e.g., Gemini with a median of 365.5) does not guarantee a successful answer (e.g., also Gemini with 89.15%).

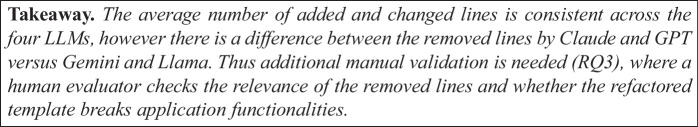



### Fix Evaluation

Finally, we evaluated each LLM refactoring in terms of whether it satisfied the tool’s policy or not. We did this by running again the tool on the refactored snippet and checking whether the alert would still be raised or not. It is important to note that even if the policy is satisfied, it does not mean that the fix is correct, because there might be false negative or positive in the tool’s results; that is why we analyzed a subset of the results manually in §7.

Table 12 provides a global view of the performance of each LLM by comparing percentages of satisfying or not the tool’s policies, not answering, or giving an answer with a wrong syntax.

**Table 12:** LLMs answers comparison across all queries

		Claude	Gemini	GPT	Llama
Total Queries		229,183	7,206	229,183	7,206
Alert not raised		58%	78%	83%	76%
Alert raised		38%	10%	16%	11%
No_Answer		1%	11%	1%	11%
Wrong syntax		2%	2%	0%	2%

Compared to Claude, Gemini, and Llama, GPT performs best when only considering the automated response by the static tools (i.e., the generated refactoring satisfies the tool policies and they do not raise any more alerts). However, this does not imply that the refactoring is correct, as shown by the results in §7 (RQ3).

Table 13 shows a comparison between the four LLMs for the top ten most common alerts, based on the structure of Table 9.

(We updated the table italic description accordingly.)

**Table 13:** LLMs answers comparison for the top ten most common alerts

ID	# Tools	% Charts	LLM Response	Claude	Gemini	GPT	Llama
26	5	90%	Alert not raised	36%	73%	85%	69%
			Alert raised	59%	19%	15%	15%
32	3	90%	Alert not raised	53%	42%	4%	40%
			Alert raised	46%	50%	95%	47%
31	5	89%	Alert not raised	48%	89%	72%	82%
			Alert raised	51%	2%	27%	6%
48	1	88%	Alert not raised	25%	10%	3%	10%
			Alert raised	74%	86%	97%	83%
49	1	88%	Alert not raised	25%	14%	3%	9%
			Alert raised	74%	83%	96%	82%
40	4	88%	Alert not raised	71%	87%	96%	78%
			Alert raised	28%	3%	3%	4%
13	5	87%	Alert not raised	39%	56%	53%	54%
			Alert raised	57%	28%	46%	26%
22	7	86%	Alert not raised	72%	83%	89%	79%
			Alert raised	23%	3%	10%	8%
27	7	83%	Alert not raised	72%	79%	91%	77%
			Alert raised	22%	3%	8%	8%
34	5	82%	Alert not raised	19%	76%	83%	84%
			Alert raised	79%	0%	16%	5%

For each row representing one of the top ten mapped policies across all charts (Table 9), this table the corresponding ID, how many tools support that policy, in how many charts it was found (in percentage), and, for each LLM, how many times the generated answer satisfied the tool, i.e., alert not raised, or vice-versa, i.e., alert raised, also in percentages. The remaining answers to arrive at 100% in each column are due to various failures, such as API and network errors, and syntax errors

Across all policies, LLMs seem to perform better for one-line fixes, i.e., 22 (Missing privilege escalation prevention), 27 (Missing read-only fs), and 31 (Missing SecComp profile), rather than policies that required new objects, such as 48 (Missing LimitRange), and 49 (Missing ResourceQuota). Interestingly, the policy most satisfied is 40 (Missing Network Policy), even though in cloud environments it is a known challenge to generate network policies (Minna and Massacci 2024). Considering those four policies (40, 22, 27, and 31), GPT seems to be providing better refactorings.

Even though specifying an AppArmor and SecComp profile in a K8s configuration takes one or few lines, and LLMs seem to perform well in this task, defining in practice one such profile is another known challenge, that requires static and runtime code and container analysis. Therefore, such LLM fixes should be considered as *shallow* fixes, where most of the work is still left to do. A similar discussion also applies to capabilities; without running the container, it is hard to estimate which are needed or not.

The three policies for which LLMs did not provide an answer most often are 34 (Minimize capabilities), 13 (Using privileged UIDs), and check_29 (Missing read-only fs). In this case, Gemini does not provide an answer most often. However, as previously already discussed, the LLM not answering may be due to external errors, such as network and API failures.

Finally, there are only three check IDs for which LLMs provided an answer with the wrong YAML syntax, namely, 26 (Using default namespace), 27 (Missing read-only fs), and 13 (Using privileged UIDs). In this case, Gemini and Llama generate the wrong syntax answers most often. However, in such cases, it is not trivial to determine the reason, thus further manual analysis is needed.

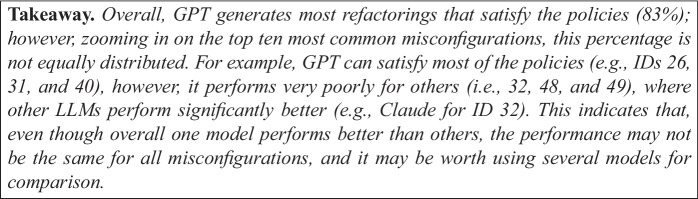



## RQ3: Manual Analysis

### Analysis Metric

To select a sample from the total population of alerts (both for the standard and smaller datasets), for the manual validation, we used the Agresti-Coull-Wilson method (NIST 2024). This method builds on the Cochran formula, which is used to calculate an ideal sample size given a defined level of precision (or margin of error), confidence level, and the proportion of the population estimated to be correct, and make inferences about all alerts. The method of Agresti and Coull improves this latter by taking into account small sample sizes and a different estimate of the population’s proportion.

The standard Cochran formula is the following:

$$n_0 = \frac{Z^2 p (1-p)}{E^2}$$

where $n_0$ is the initial sample size, *Z* is the desired confidence level, *p* the estimated proportion of the population, and *E* the desired margin of error.

The adjusted proportion *p* using the Agresti and Coull is calculated as follows:

$$p\prime = \frac{x + 2}{n + 4}$$

where *x* is the number of successes in the sample, and *n* is the sample size.

For our experiment, for the standard dataset, we selected $Z = 1.96$ (i.e., 95% confidence), $p = 50\%$ and $E = 10\%$, whereas for the smaller dataset, we selected $Z = 1.96$ (i.e., 95% confidence), $p = 10\%$ and $E = 10\%$. This corresponded to 90 randomly selected charts for Claude and GPT (standard datasets), and 30 for Gemini and Llama (smaller datasets).

By using such a formula we can perform the manual analysis on a smaller sample than the entire dataset and project the findings of this latter on the entire population, with a predefined confidence interval and error rate; in fact, this lets us avoid manually checking every item in each dataset, which would be extremely time-consuming and error-prone.

### Evaluation Procedures

We evaluated 30 charts for Gemini and Llama (smaller dataset), and 90 charts for Claude and GPT (standard dataset); each chart was randomly selected using the numpy random.randint function.

First, we introduce the rules by which we determine the correctness of the tool output and LLM refactorings. Because we have two operands, i.e., the tool’s output (alert raised or not) and policy state (satisfied or not), the total number of combinations (the order is not relevant) is $2 \cdot 2 = 4$. Between the two operands, there is always the *and* logic operator ($\wedge$), therefore we have four possible rules and each rule can generate a result, that can either be correct or wrong; for example, if $operand\_1 = True$ and $operand\_2 = True$, then $result = wrong$. In our case, given a policy, its state can be determined by manually looking at the YAML code; instead, the tool’s output is always generated by analyzing the YAML code with the tool. Also, we can assign a metric to each rule’s result, specifically, true negative (TN), false positive (FP), true positive (TP), and false negative (FN).

Table 14 shows the combination of such four rules, along with the corresponding result and metric; each column represents a rule, whereas the rows are respectively the first operand (policy state), the operator (always $\wedge$), the second operand (tool’s output), and the corresponding result.

**Table 14:** Rules to determine the correctness of the results

	Rule 1	Rule 2	Rule 3	Rule 4
Policy State	Satisfied	Satisfied	Not Satisfied	Not Satisfied
Tool Output	No Alert	Alert	Alert	No Alert
Result	Correct	Wrong	Correct	Wrong
Metric	TN	FP	TP	FN
Comment	No misconfiguration present, and no alert raised.	No misconfiguration present, but alert raised.	Misconfiguration present and corresponding alert raised.	Misconfiguration present, but alert not raised.

The policy state can be manually determined by looking at the YAML code whereas the tool’s output is always generated by analyzing the YAML code with the tool. The four metrics are, respectively, true negative (TN), false positive (FP), true positive (TP), and false negative (FN)

We used those rules to determine the tools’ and LLMs’ correctness by manually evaluating the policy state on the original resource and LLM snippet YAML code and generating a tool’s output for each code for each query. Because all queries were generated from all tool’s alerts, for the tools, we only evaluated the FP and TP rates (i.e., *Rule 2* and *Rule 3*); if no alert was raised (i.e., *Rule 1* and *Rule 4*), no query was generated. Therefore, we did not have a case where we asked the LLM to fix a correct configuration.

Table 15 shows the application of the four rules previously defined (Table 14) on both the original resource and LLM snippet YAML code, to determine the tool’s and LLM’s correctness.

**Table 15:** Application of the rules to determine the correctness of the tool and LLM results

		Applicable rules			
Input	Subject	Rule 1	Rule 2	Rule 3	Rule 4
Original Resource	Tool	-	Wrong (FP)	Correct	-
	LLM	-	-	-	-
LLM Snippet	Tool	Correct	Wrong (FP)	Correct	Wrong (FN)
	LLM	Correct	Correct	Wrong (no fix)	Wrong (no fix)

For the Original YAML, Rules 1 and 4 are out of scope for this paper, because they would require manually looking at the YAML code even if no alert was raised; similarly, for the Original YAML, all rules are also out of scope for the LLM, because this would correspond to using the LLM to detect misconfigurations

Algorithm 2 shows the pseudocode of the application of the four rules (Table 15 and Table 14) on both the original resource and LLM snippet YAML code, to determine the tool’s and LLM’s correctness.

**Algorithm 2:**
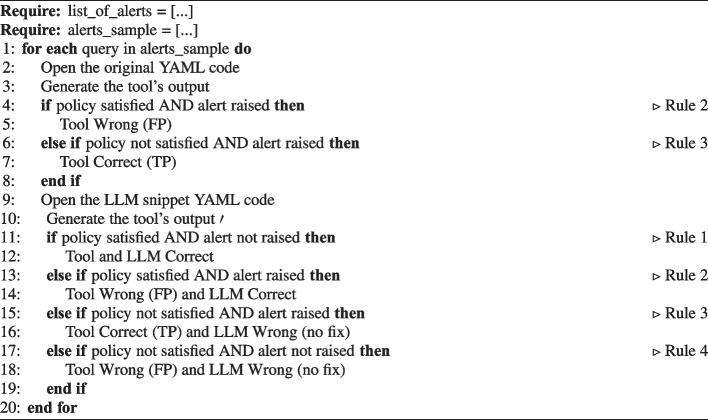
Procedure to manually evaluate the four rules on the original resource and LLM snippet YAML code.

Finally, Algorithm 3 shows the procedure that we used to evaluate the changed lines in the LLM refactored output, compared to the original code; in fact, in some cases, the LLM can satisfy the policy by adding a good fix and adding and removing irrelevant additional lines.

**Algorithm 3:**
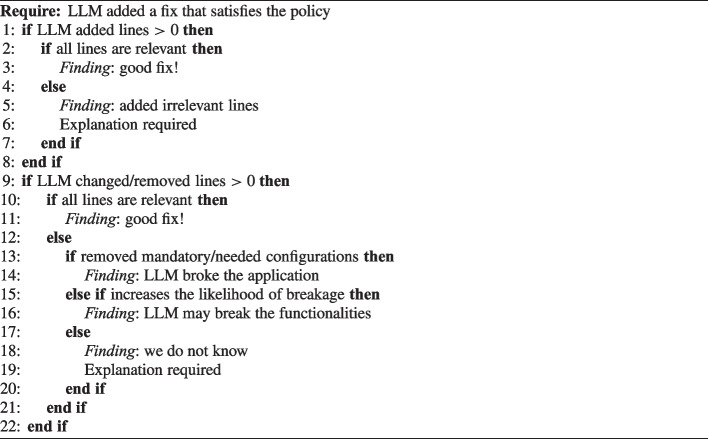
Pseudocode of the algorithm to manually evaluate LLM line changes.

The evaluation results of applying such algorithms are reported in the next subsection.

### Evaluation Results

As previously explained, for RQ3, the ground truth is computed by manually evaluating the YAML snippet generated by the LLMs, using Algorithm 3, and the results are reported in the confusion matrix, both for the LLM snippet and Merged Resource, with the four metrics TN, FP, TP, and FN.

Table 16 shows the metric values for each resource we manually analyzed. Specifically, the “Original Resource” corresponds to the chart template downloaded from Artifact Hub as is; as explained before, because all queries were generated from all tool’s alerts, for the tools, we only evaluated the FP and TP rates (i.e., *Rule 2* and *Rule 3*). Instead, the “LLM Snippet” corresponds to the LLM’s response by *only* considering lines relevant to the fix, ignoring everything else; instead, “Merged Resource” corresponds to the merge of the LLM snippet into the original resource. This distinction is important because LLMs can add, change, and remove additional irrelevant lines, besides the lines of the fix, which we must consider.

**Table 16:** Metrics (TN, FP, TP, and FN) results for the original resource, LLM snippet, and merged resource

LLM	# Charts	LLM Snippet				Merged Resource			
		TN	FP	TP	FN	TN	FP	TP	FN
Claude	90	82%	0%	13%	0%	82%	0%	10%	3%
Gemini	30	77%	0%	10%	7%	47%	0%	13%	20%
GPT	90	77%	0%	14%	1%	71%	0%	14%	4%
Llama	30	60%	0%	10%	13%	33%	0%	20%	33%

The original resource is the original chart YAML code, the LLM snippet is only the code lines relevant to the fix, and the merged resource is the merge of the LLM snippet into the original resource. The sum may be lower for each metric than the total number of charts due to Python parsing errors or deprecated policies, which we did not count in the final results

We used the following seed values to generate the sample of charts for each LLM, namely, 2, 410 for Claude, 94, 516 for Gemini, 75, 551 for GPT, and 30048 for Llama, randomly generated with the numpy.random.randint function.

For the original resource, we found all the alerts to be correct, thus with the reported misconfiguration present ($FP = 0$). This indicates a certain reliability and robustness by such tools in detecting different misconfiguration types in several templates. However, we found some false negative results, both for the LLM snippet and the merged resource; this is likely because in the query we do not always provide the whole original resource, but a snippet where the tool indicated there is a misconfiguration, as explained in §4.6. In some cases, the location corresponds to the all resource, whereas in other cases to a snippet; in this latter case, the tools may not raise an alert, because they do not recognize it as a K8s valid resource.

When considering only the lines of the fix (LLM snippet), Claude seems to be the LLM that performs better (82% of TN), followed by Gemini and GPT; when considering the merged resource (LLM snippet + original resource), Claude is still the best LLM, followed by GPT, whereas Gemini and Llama perform worst.

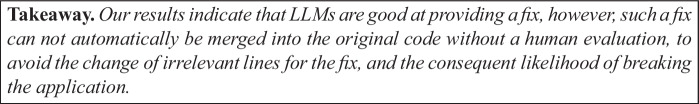



For the snippets selected for the manual analysis, based on Table 12, we also investigated whether the fixes added by the LLM introduced other issues. Table 17 shows the percentages of each LLM when they fixed the misconfigurations without introducing issues (“Alert not raised”) and viceversa, when they did not fix the misconfigurations (“Alert raised”), and answers with a wrong syntax.

**Table 17:** LLMs answers extensive comparison across all queries

	Claude	Gemini	GPT	Llama
Total Queries	90	30	90	30
Alert not raised	82%	70%	77%	50%
Alert not raised with issues	0%	10%	4%	10%
Alert raised	13%	13%	11%	27%
Wrong syntax	4%	3%	8%	7%

We removed the No_Answer row because we did not consider those queries for the manual analysis. The total may be less than 100% because we excluded false positive alerts from the tools

Only in few cases LLMs removed a misconfigurations introducing new issues or wrongly changing the original code. For example, Gemini for one query added a correct fix but deleted other relevant configurations (e.g., dropping the capabilities and running as not root), thus providing a good fix but overall making the configuration more insecure. In other cases, the LLM added a fix for other misconfigurations rather than the one specified in the query; in such cases, spending more effort into the query engineering process may address the issues. Such cases strengthen our claim that LLM can be used to provide suggestions for safer configurations, however, the answer must always be reviewed by a human because, even for good fixes, the model may introduce additional issues.

To further investigate each LLM performance, we looked into the TN and FN metric values for both the LLM snippet and merged resource; we excluded TP results, which are known to be wrong, and FP results, which we never found. Therefore, with the FN results found during the manual analysis, we computed the apparent fix rate percentage, when considering TN + FN as the chart sample, and the true fix rate percentage when considering the original sample size. Table 18 shows the apparent and true fix rate of each LLM for the LLM snippet.

**Table 18:** LLMs fix rate for the LLM snippet

LLM	# Charts	LLM Snippet			
		TN	FN	Apparent Fix Rate	True Fix Rate
Claude	90	74	0	100%	82%
Gemini	30	23	2	92%	70%
GPT	90	69	1	99%	76%
Llama	30	18	4	82%	47%
		# charts	# charts	TN/(TN+FN)	(TN-FN)/# charts

Similarly, Table 19 shows the apparent and true fix rate of each LLM for the merge.

**Table 19:** LLMs adjusted fix rate on the merged resource

LLM	# Charts	Merged Resource			
		TN	FN	Apparent Fix Rate	True Fix Rate
Claude	90	74	3	96%	79%
Gemini	30	14	6	70%	27%
GPT	90	64	4	94%	67%
Llama	30	10	10	50%	0%
		# charts	# charts	TN/(TN+FN)	(TN-FN)/# charts

For the LLM snippet, when considering the TN and FN results over the sample size (i.e., the number of charts), there is a drop in performance for all the LLMs. Claude is still the best LLM, whereas Llama’s fix rate drops to 47%, meaning that in half the queries you can get a wrong answers.

For the merged resource, performances drop even more significantly. Claude remains the best, followed by GPT, Gemini drops to around one correct answer every four queries, whereas Llama fix rate drops to 0, generating a wrong refactoring for each query. The reason for Gemini and Llama to perform in the worst way may be due to the changes that they make to lines irrelevant to the fixes; in fact, when considering the removed lines metrics (shown in Table 11), these two LLMs remove a significantly higher number of lines compared to Claude and GPT.

According to these results, we found the best LLM for fixing Helm chart misconfigurations to be Anthropic Claude-3-sonnet.

During our manual evaluation, we only found false positive results for one policy of one tool (“Missing AppArmor profile” in KICS), due to the policy checking a deprecated K8s configuration, which we did not consider in the fix rate percentage computation.

Finally, Table 20 shows the results of the procedure described in Algorithm 3 during the manual analysis, related to whether LLMs added, changed, or removed irrelevant lines for the fixes, and whether LLMs also fixed the YAML syntax or not.

**Table 20:** The results of the Algorithm 3 during the manual analysis

LLM	# Charts	No Added Irrelevant	No Changed/Removed Irrelevant	Correct Original Syntax	Correct Refactored Syntax
Claude	90	92%	93%	80%	91%
Gemini	30	87%	80%	80%	93%
GPT	90	80%	91%	87%	97%
Llama	30	77%	57%	73%	77%

We only evaluated the irrelevant lines and the syntax either when the LLM provided a good fix (TN) or when the tool did not detect a misconfiguration after the refactoring (FN)

Regarding the irrelevant lines, Claude did not add, change, or remove irrelevant lines in any of the evaluated charts; the missing percentages are due to other errors, such as the Python YAML library. Gemini, in two refactorings, added irrelevant configurations, specifically, dropping all capabilities and runAsNonRoot, and once deleted irrelevant lines; in such cases, we counted as “likely broken” because it is hard to evaluate without deploying the chart. In one very interesting case, it added a good fix (read-only filesystem), but deleted other safe irrelevant configurations, such as drop capabilities, and run as not root; in this case, even though it did not break the configuration, it made it more insecure. GPT, instead, for one policy (allowPrivilegeEscalation) always added runAsNonRoot as well, likely breaking the configuration, if the containers need the root user to run; only in one case, it deleted many irrelevant lines. Finally, Llama is the model performing the worst, changing and deleting irrelevant lines in almost 50% of the queries. The common behavior that we observed in Llama refactorings was adding a good fix and then deleting most of the following lines; this may indicate that the model is trying to save on the tokens being used, however, by doing so, generating wrong answers.

Regarding the syntax, all models, besides Llama, showed very good performances in removing syntax errors; this may indicate that LLMs can fix and generate syntactically correct code.

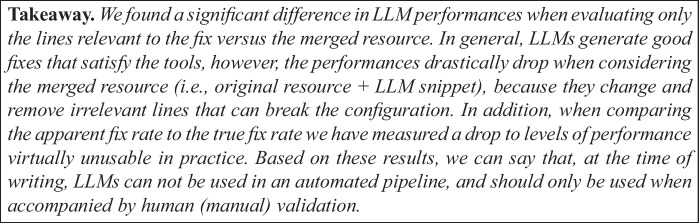



## General Results Discussion

Finally, after answering the three RQs, we present a general discussion of the evaluation, results, and findings about the tools and LLMs.

By answering RQ1, we found that there was at least one misconfiguration in most of the charts. Also, most tools do not check for YAML syntax, so it may be worth it to use another tool or one that supports it, e.g., Datree. Regarding the top ten misconfigurations that we found, the first six are either very cloud environment dependent, such as the workload namespace, LimitRange, and ResourceQuota, or know cloud security challenges, such as defining AppArmor and SecComp security profiles, or network policies. Generating a fix for these latter three policies may require static and run-time source code analysis, and container deployments, thus an LLM can be out of scope for this.

When querying the LLMs for a fix, we found that the number of tokens being used is not related to the correctness of the answer; for example, Gemini, on average, uses more input and output tokens compared to the other LLM but does not perform better. Instead, there is a significant difference between the number of removed lines, which is a clear need for additional manual validation of the model outputs.

Also, when evaluating the LLM that provides the best answers (i.e., GPT, according to the analyzer tools), this performance is not equally distributed; for example, GPT performs very well on some policies, but very poorly on others, suggesting that using at least two models for comparison would lead to better results.

Finally, during our manual evaluation, we found good robustness of the tools in finding misconfigurations across different templates; even though a more extensive analysis of the reported misconfigurations is needed, we did not find any false positive or negative results, supporting the correctness of the misconfiguration reported in RQ1. Regarding the LLM refactorings, we found that LLMs can be useful and perform best when prompted to provide simple fixes (i.e., of very few lines). For complex fixes or large inputs of several lines, the LLMs can also change or remove lines irrelevant to the fix; for example, for an input of 200 lines, the LLM can provide a correct fix at line 50, shortening or removing the following lines to save tokens. Therefore, even though the fix is correct, the answer can not automatically be merged into the original code, because this can affect also irrelevant lines, likely breaking the configuration.

Finally, based on the results of this paper, we give some recommendations to LLM users, especially those who aim to use such models to generate new configurations or refactor and improve existing ones. We found that giving as input in the query a snippet of the resource, instead of all Deployment configurations or templates, will not make a difference in most cases. This can reduce the costs (less code — fewer tokens), make it easy to manually evaluate the answer, and less likely for the model to change irrelevant lines. Also, our results suggest that LLMs perform best to fix simple misconfigurations (few lines); thus, providing the snippet can be enough. In the case of a model failing to answer, we suggest to re-query again, without any change; in fact, the failure can be due to network, APIs, or the model’s internal errors, so requiring can likely make the model generate an answer. Finally, we strongly suggest manually evaluating the model answer and manually merging it into the original code. By doing this, it is possible to both evaluate the correctness of the answer and avoid merging into the original code irrelevant lines, besides the fix, or, even worse, changing or removing existing configurations.

## Threats to Validity

### Internal Validity

The results of this paper are computed based a large fraction of all publicly available charts on Artifact Hub; however, private charts or charts deployed by companies in private clusters might yield different results. To compute and analyze such results, we developed our own Python scripts, for example, to parse the JSON tool output generated file, read and save data into CSV files, query LLMs through the APIs and save the response, count the changed lines, etc. Errors and bugs in the implementation and Python libraries may also generate wrong results and throw exceptions; also for this latter reason, we used the manual analysis to verify the correctness of our implementation. We also never deployed the charts in a K8s cluster; if, in some cases, determining broken configurations is trivial (e.g., a missing image in a Pod), in other cases it can not be determined at static time without a deployment (e.g., container without root user). In future work, it can be interesting to complement this static analysis by also deploying such configurations.

### External Validity

To evaluate each chart, we only considered a set of seven open-source chart analyzer tools, however, other tools (such as proprietary tools) might also yield different results. During the time of writing new tools may emerge in the market, such as Fairwinds (2024), therefore the overall results may also differ. It is well known that the quality and correctness of the LLM answers largely depend on the query engineering process; in our case, we did not do a study and evaluation on this process and for this reason, we used the policy description provided by each tool. However, a query engineering study can improve both the refactoring results and the quality of the generated configurations in terms of misconfigurations removed; this can be an interesting starting point for future work.

### Construct Validity

We assumed that malicious information and misconfigurations had not been inserted into the chart templates on purpose and that analyzer tools had not been compromised and the results were correct; however, eventual errors or bugs might also yield different results. We partially mitigated the risk of the tools being compromised by doing a manual analysis of the results, finding 0% of false positive alerts.

## Conclusion and Future Work

In this experiment, we mined and empirically evaluated the security of all Helm charts available on Artifact Hub. First, we used seven state-of-the-art analyzer tools to find the most common misconfigurations, i.e., using default namespace, and missing SecComp and AppArmor profiles; then, we used four LLMs, namely, GPT-4o-mini-2024-07-18, Google Gemini 1.5, Anthropic Claude-3-sonnet, and Meta Llama3-70b, to remove such misconfigurations. In terms of correctness, Claude is the LLM performing best, however, the takeaway of this research is that, due to the low correctness percentage of generated answers, such models can not be used in an automated pipeline, and must be accompanied by manual evaluation. They can be effective in generating simple (one or two lines) fixes, however, they can also change and remove irrelevant lines, thus breaking the configuration; that is why a human evaluating the answer and manually merging this latter into the original code is critical.

For the future work, LLM failures should be further investigated under several aspects, e.g., model failures and query engineering, because we believe they are interesting research directions and can provide several insights. For example, it should be investigated whether certain queries make the model fail versus other queries, and to what extent query engineering can improve the configuration fix rate. Other failures, such as network and model related, should be further investigated, as well as trying to re-query a model upon failure. Finally, it can be interesting to evaluate LLM capabilities in terms of detecting misconfigurations, and not only fixing them.

To conclude, as authors of the registered report track, we found this approach very useful and effective. The main benefit was early feedback and an initial review on the proposed study’s method and hypothesis. In fact, we believe this approach saved a lot of time to the authors, by making sure the study is based on a strong method and it is different and new from the state of the art, and formulate hypothesis that are interesting to the community. Having the opportunity to present the initial report at the conference, further gives the opportunity to network with other interested researchers and improve the study along the execution, or as future work.

## Data Availability

The Python scripts source code used to download the Helm charts, run the analyzer tools, query the LLMs, and save the results, are available at the following GitHub repository: https://github.com/fminna/LLM-Helm-Fix. Data supporting the answers to each research questions is available as CSV files at the following Zenodo repository: https://doi.org/10.5281/zenodo.14294639

## Appendix CIS Benchmark Clusters Policy Mapping

Table 21 shows the mapping between each policy ID to the corresponding CIS Benchmark Cluster, along with how many tools support the policy and a brief description.

The table in A1 does not mention the top found policies in the body of the paper on Table 9. E.g. 13 is not there.

**Table 21:** Mapping between policies and CIS BenchMark Clusters

ID	# Tools	Description	CIS Cluster
10	7	Sharing host’s PID ns	Pod Security Policy
11	6	Sharing host’s IPC ns	Pod Security Policy
12	7	Sharing host’s network ns	Pod Security Policy
21	6	Privileged container	Pod Security Policy
22	7	Missing privilege escalation prevention	Pod Security Policy
23	5	NET_RAW capability	Pod Security Policy
24	4	SYS_ADMIN capability	Pod Security Policy
26	5	Using default namespace	General Policies
28	7	Root container	Pod Security Policy
30	6	Missing securityContext	General Policies
31	5	Missing SecComp profile	General Policies
33	5	Secrets as files instead of env var	Secrets Management
34	5	Minimize capabilities	Pod Security Policy
35	6	Mounted Service Account Token	RBAC and Service Accounts
36	3	Default Service Account used	RBAC and Service Accounts
37	3	Undefined Service Account name	RBAC and Service Accounts
39	2	Wildcard in Role and ClusterRoles	RBAC and Service Accounts
40	4	Pod without NetworkPolicy	Network Policies and CNI
54	4	Mutating admission webhook configurations	RBAC and Service Accounts
59	1	ServiceAccount can read secrets	RBAC and Service Accounts
70	1	Namespace without NetworkPolicy	Network Policies and CNI
77	1	Role/ClusterRole bonded to default Service Account	RBAC and Service Accounts
78	1	Role/ClusterRole with port-forward permission	RBAC and Service Accounts
80	1	Role/ClusterRole with attach to containers permission	RBAC and Service Accounts

## Footnotes

1 https://www.cisecurity.org/cis-benchmarks

2 https://artifacthub.github.io/hub/api/

3 https://ai.google.dev/tutorials/python_quickstart

4 https://hub.datree.io/built-in-rules/prevent-escalating-privileges

5 https://hub.datree.io/built-in-rules/ensure-memory-request
